# Functional imaging of cognition in an old-old population: A case for portable functional near-infrared spectroscopy

**DOI:** 10.1371/journal.pone.0184918

**Published:** 2017-10-12

**Authors:** Theodore J. Huppert, Helmet Karim, Chia-Cheng Lin, Bader A. Alqahtani, Susan L. Greenspan, Patrick J. Sparto

**Affiliations:** 1 Department of Radiology, University of Pittsburgh, Pittsburgh, Pennsylvania, United States of America; 2 Department of Bioengineering, University of Pittsburgh, Pittsburgh, Pennsylvania, United States of America; 3 Department of Physical Therapy, East Carolina University, Greenville, North Carolina, United States of America; 4 Department of Electrical Engineering, University of Pittsburgh, Pittsburgh, Pennsylvania, United States of America; 5 Department of Medicine, University of Pittsburgh, Pittsburgh, Pennsylvania, United States of America; 6 Department of Physical Therapy, University of Pittsburgh, Pittsburgh, Pennsylvania, United States of America; University of California, San Francisco, UNITED STATES

## Abstract

In this study, functional near-infrared spectroscopy (fNIRS) was used to record brain activation during cognitive testing in older individuals (88±6yo; N = 19) living in residential care communities. This population, which is often associated with loss of personal independence due to physical or cognitive decline associated with aging, is also often under-represented in neuroscience research because of a limited means to participate in studies which often take place in large urban or university centers. In this study, we demonstrate the feasibility and initial results using a portable 8-source by 4-detector fNIRS system to measure brain activity from participants within residential care community centers. Using fNIRS, brain signals were recorded during a series of computerized cognitive tests, including a Symbol Digit Coding test (SDC), Stroop Test (ST), and Shifting Attention Test (SAT). The SDC and SAT elicited greater activity in the left middle frontal region of interest. Three components of the ST produced increases in the right middle frontal and superior frontal, and left superior frontal regions. An association between advanced age and increased activation in the right middle frontal region was observed during the incongruent ST. Although none of the participants had clinical dementia based on the short portable mental status questionnaire, the group performance was slightly below age-normed values on these cognitive tests. These results demonstrate the capability for obtaining functional neuroimaging measures in residential settings, which ultimately may aid in prognosis and care related to dementia in older adults.

## Introduction

In 2014, approximately 830,000 older adults in the US lived in a residential care community (RCC), which is one type of long-term care facility [1]. Residential care communities serve individuals with cognitive impairment, dementia, or Alzheimer’s disease. In 2010, about 60% of residents had some form of cognitive impairment, consisting of 18% without dementia and 42% with Alzheimer’s disease or other dementia [2]. These residents with cognitive impairment had more emergency department visits, required greater assistance with activities of daily living, greater care for urinary incontinence, and more skilled nursing care, resulting in an additional $7,000 of care per year. The overall cost for individuals with cognitive impairment residing in RCCs was $17 billion [2].

About 6% of RCCs are part of facilities that provide a continuum of care that include skilled nursing, assisted living, independent, and dementia units [2]. Residents often transition from one level of care to another depending on acute illness, cognitive status, family requests or physician input/supervision. Consequently, characterization of the health status of individuals living in these facilities is better-established using functional status rather than the specific setting that they currently reside in. Therefore, assessment of cognitive function using simple, portable tools may provide some benefit for facilities to provide the optimum care and manage costs.

Assessment of cognitive function, specifically executive function, is relatively easy to perform using the traditional paper-and-pencil format, or newer computer-based formats [3–5]. In addition to the overall performance results that may reveal deficits in different executive functions, it is possible that additional important information about the cognitive status may be obtained from the functional brain activation as individuals are performing the tests [4, 6–9]. However, research in this age group is somewhat limited by a well-acknowledged selection bias towards individuals with the means and motivation to travel to urban and college/university centers where the majority of research using magnetic resonance imaging (MRI) or other neuroimaging methods are performed. Functional near-infrared spectroscopy (fNIRS) is a non-invasive neuroimaging technology that uses low levels of red to near-infrared light (650-900nm) to spectroscopically measure hemoglobin changes in tissue. Biological tissue has low intrinsic absorption in this range of wavelengths, which allows light to remain detectable even after it has passed through up to centimeters of tissue. However, in this range, tissue is highly optically turbid, which means that the light scatters and changes directions multiple times, creating a diffuse path through the tissue along which the optical properties are examined. Functional NIRS instruments record the changes in the optical absorption of the tissue along this diffuse path, for example, due to an increase in regional blood flow, which differentially absorbs light due to the presence of oxy- and deoxy-hemoglobin. A grid of light sources and light detectors are used to spatially localize the change in optical frequency between each source-to-detector measurement pair.

In an fNIRS study, the participant wears a head cap embedded with light sources and detectors positioned over the specific part of the brain under investigation. During cognitive performance, regional neural activity causes a change in the blood flow, volume, and oxygenation of the brain through mechanisms of neural-vascular and neural-metabolic coupling. The fNIRS source-detector pairs measure the light transmitted through the region of interest and infer brain activity via statistical analysis of the evoked changes in oxy- and deoxy-hemoglobin concentration. The depth of penetration into the brain is limited to the outer the cortex of the brain, depending on the source-detector spacing [10]. Although this is more superficial than other modalities such as functional magnetic resonance imaging (fMRI), this still allows for measurements from most cognitive regions of the cortex. Previously, fNIRS has been used to investigate a variety of cognitive tasks [11–16](reviewed in [17]) and has particular utility in child populations where more traditional MRI methods are difficult (reviewed in [18]). Consequently, the use of fNIRS for investigating cognitive function in a setting outside of the laboratory, such as in RCCs, may be a useful application. A limitation of fNIRS in this context, however, is that because these measurements are recorded from sensors placed on the surface of the scalp, the sensitivity to specific brain regions depends on individual variation in the anatomy of the head and is therefore affected by factors such as atrophy and head size.

In this study, we used portable fNIRS to measure brain activation during cognitive tasks in older adults (65 or older) living in RCCs. The purpose of this study is to establish the viability of using a portable fNIRS device in populations that otherwise have barriers to participating in typical brain imaging studies. Specifically, subjects living in RCCs are under-represented due to limitations in access to studies that use scanners that are fixed in a location (e.g. MRI, PET, MEG). In order for very old subjects to participate in traditional brain imaging studies, they need to be able to get to and from the scanner facilities. Thus, subjects over 65 that do participate in these studies tend to be healthier and in a higher socioeconomic status than the representative population. This paper focuses on our methods for portable fNIRS data collection and analysis. We hypothesized that, by using well-established tests of cognition, we would be able to find a significant result of activation, thus indicating that portable fNIRS is a worthwhile method of recording brain activation in the very old.

## Materials and methods

### Subject population

Participants were independently living in long-term care facilities, specifically RCCs, defined as facilities that provide room, board with at least two meals a day, and help with personal care such as bathing and dressing or health-related services, such as medication management [1]. Nineteen participants (13 F, 88.1 ± 6.0 years) were enrolled in this research study. All subjects were recruited using a convenience sample of older adults living in three RCC facilities, who were already enrolled in a research study investigating the mobility and gait function of residents in RCC settings [19]. The study was approved by the Institutional Review Board of the University of Pittsburgh. The study included individuals who were aged 65 years or older and cognitively able to provide informed consent, initially based on reports from resident staff and later confirmed with the Short Portable Mental Status Questionnaire [20]. Gait speed was measured as the average of two 4-meter self-paced walks. Because this study was part of a larger one that investigated balance and strength in this population, individuals were excluded if they were not able to ambulate for 1 minute (assistive devices were allowed), or had any medical or neurological condition that prevented them from performing maximal muscle action. The medical history was assessed using an 18-item comorbidity index and number of prescription medications used, but these were not used in analysis since the initial goal of this study was to show pilot feasibility of the use of fNIRS in this population (Table 1).

**Table 1:** Indicators of health status, including number of comorbidities, number of prescription medications, and cognitive function (Short Portable Mental Status Questionnaire, SPMSQ).

Subject Number	Comorbidities (n)	Medications (n)	SPMSQ (total errors)
**1**	8	2	1
**2**	3	5	0
**3**	8	9	0
**7**	13	14	1
**9**	8	9	1
**12**	6	3	2
**13**	4	5	1
**17**	11	6	1
**19**	4	5	0
**21**	5	4	0
**24**	8	3	2
**25**	9	3	0
**26**	8	5	0
**27**	10	11	0
**31**	7	9	2
**51**	5	10	1
**53**	8	10	1
**56**	7	14	0
**65**	6	9	2

Any SPMSQ score 2 or below indicates normal cognitive functioning.

### Instrumentation

**Fig 1:**
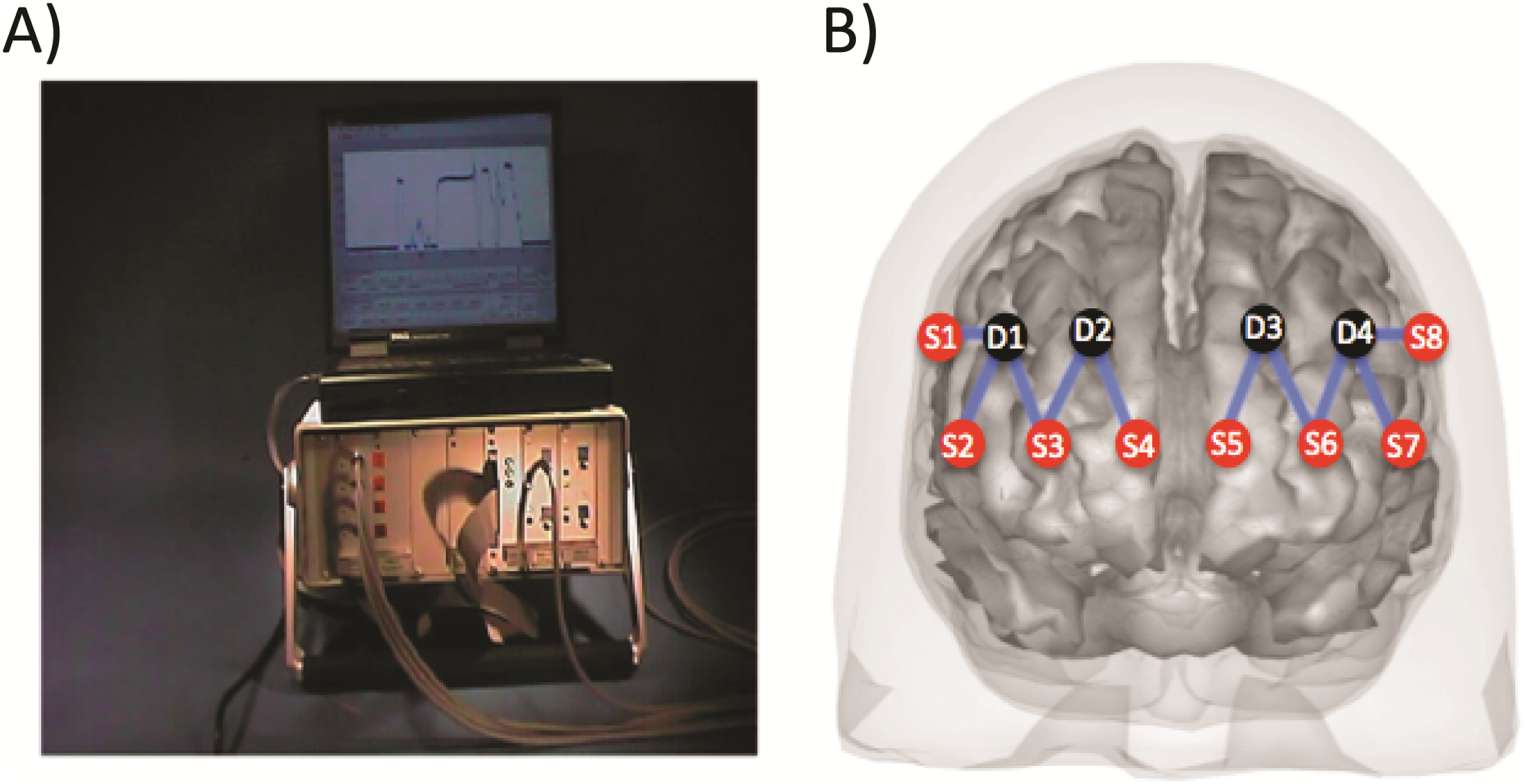
Functional NIRS setup. The photograph (1A) shows the TechEn NIRS-2 instrument, which was connected via fiber optic cables to the fNIRS head probe. The registered location of the fNIRS head probe is shown (1B) and consisted of 8 source and 4 detector positions (32mm separation) positioned bilaterally across the forehead.

A portable 4 source x 4 detector fNIRS system (NIRS-2, TechEn Inc, Milford MA USA; Fig 1A) was used in this study. This instrument used four laser diode sources at 808nm and four avalanche photodiode detectors. We used 808nm as this is an isobestic point of hemoglobin, where both oxygenated and deoxygenated forms absorb light similarly. At this wavelength, fNIRS measurements of tissue optical absorption between pairs of source and detector positions reflect underlying changes in total-hemoglobin as an analog of cerebral blood volume. Although fNIRS is typically recorded at two wavelengths in order to distinguish changes in the oxygenated and deoxygenated forms of hemoglobin, the use of the single isobestic measurement in this study allowed us to double the number of spatial positions on the head and was a compromise given the portability constraints of this field study. A limitation of this, however, is that our study is limited to reporting values proportional to total-hemoglobin changes.

The fNIRS instrument used fiber optic cables to transmit light to and from the instrument and a sensor probe placed on the participant’s forehead. In order to further increase the spatial sampling, bifurcated source fibers which send a single instrument laser light concurrently to both sides of the head were used to double the number of source positions on the head allowing for a total of eight source positions (four per hemisphere; see S1and S2 Figs). A total of four detector positions (two per hemisphere) collect the light as it exits the tissue in order to record the changes in the optical absorption between source-to-detector pairs. By making these modifications to the standard 4x4 NIRS-2 instrument, we were able to record a total of 10 source-to-detector pairs on the head. Functional NIRS signals were sampled at 200 Hz and then Nyquist filtered and resampled to 20 Hz prior to analysis. The fiber optics were arranged on the forehead as shown in Fig 1B with a source-detector separation of 32 mm. A 32mm separation was used based on empirical experience from previous studies and is a compromise between depth of penetration and signal-to-noise of the measurements. In order to align the fNIRS cap, the center of the cap was adjusted to the 10–20 point FpZ, which was used for registration of the fNIRS head cap. The positions of the fNIRS sensors were then estimated relative to this point on the Colin27 atlas head [21] using custom Matlab code. The position of the fNIRS measurements (mid-point between the source-detector pair) relative to this atlas was presented in Table 2. In this study, this registration to an atlas was used for the definitions of approximate regions-of-interest in the analysis (see S2 Fig). Since individual anatomical information (e.g. structural MRI) was not available for any of the subjects, variability in head and brain anatomy is expected to contribute to underestimation of the statistical effect size of brain responses. Likewise, systematic differences in anatomy (e.g. correlation between cortical atrophy and age) would introduce bias in the fNIRS results since the sensitivity to the brain would fall exponentially as the scalp-to-brain distance increased.

**Table 2:** Location of NIRS sensors based on registration to the Colin-27 atlas.

fNIRS channel	Nearest region of interest	
Source 1: Detector 1	Right Middle Frontal	BA-46
Source 2: Detector 1	Right Middle Frontal	BA-46
Source 3: Detector 1	Right Superior Frontal	BA-45
Source 3: Detector 2	Right Superior Frontal	BA-10
Source 4: Detector 2	Right Superior Frontal	BA-10
Source 5: Detector 3	Left Superior Frontal	BA-10
Source 6: Detector 3	Left Superior Frontal	BA-10
Source 6: Detector 4	Left Superior Frontal	BA-45
Source 7: Detector 4	Left Middle Frontal	BA-46
Source 8: Detector 4	Left Middle Frontal	BA-46

### Experimental design

Functional NIRS testing was performed in an unoccupied living suite, similar to the suite in which the participants resided. Brain imaging was done while participants performed three cognitive tests as part of the computer-administered CNS Vital Signs (Morrisville, NC) test. Specifically, the Symbol Digit Coding test, Stroop Test, and the Shifting Attention Test were performed in fixed order. The tests were carried out using a laptop computer under standard procedures recommended by the manufacturer, using a custom color-coded keyboard provided by the manufacturer. Prior to each test, the subject performed a brief trial of the test so that understanding of the procedures could be confirmed. Before and after each test, 30 seconds of baseline fNIRS measurements were recorded while the subject sat quietly.

#### Symbol Digit Coding (SDC)

This test measures the speed with which subjects can mentally transform one symbol into another based on a given code. A code table is presented at the top of a monitor in which eight different symbols are arranged in a row. Beneath each symbol in the code table is a different digit [2–9]. Below the code table are rows of double boxes. In the top of each double box is a symbol while the bottom box is empty. The subject types in the appropriate digit under each code, and completes as many boxes as possible within 120s [22]. The number completed successfully is the outcome measure, which is then percentile scaled based on age-normed performance.

#### Stroop Test (ST)

The ST has three parts. In the first part, which is a simple reaction time (SRT) task, the words RED, YELLOW, BLUE, and GREEN (printed in black) appear at random on the screen, and the participant presses the space bar as soon as he/she sees the word (Stroop SRT, 30 s duration). In the second part, the words RED, YELLOW, BLUE, and GREEN appear on the screen, printed in different colors. The participant presses the space bar when the color of the word matches what the word says (Stroop Congruent, 45s duration). In the third part, the words RED, YELLOW, BLUE, and GREEN appear on the screen in different colors, as before. This time, the participant presses the space bar when the color of the word does not match what the word says (Stroop Incongruent, 90s duration) [22]. The reaction times from each subtest were used as outcomes, and percentile based on normed performance.

#### Shifting Attention Test (SAT)

The SAT test is a measure of ability to shift from one instruction set to another quickly and accurately. Participants are instructed to match geometric objects either by shape or by color. Three figures appear on the screen, one on top and two on the bottom. The top figure is either a square or a circle, in either red or blue color. The shape and color are randomly selected. The bottom figures are a square and a circle presented side-by-side in either red or blue, so that both shapes and colors are presented (e.g. a red square is paired with a blue circle). The matching rule is displayed above the top figure (i.e. “Match SHAPE”, “Match COLOR”). The participant matches one of the bottom figures to the top figure based on the rule by pressing the left or right shift key. The rule is randomly selected [22]. This test lasted for approximately 90s. The raw and percentile reaction time and number correct were used as outcomes of this test and are normative for age.

### Analysis methods

Functional NIRS data was analyzed using a general linear model to detect measurement channels that were statistically related to the timing of the stimulus events. In brief, the fNIRS raw signals (light transmitted between each source-to-detector pair) were converted to a measure of the change in optical absorption over time for each pair. At the 808 nm wavelength, this measurement is proportional to total-hemoglobin changes. The time course for each pair per scan was then analyzed using a general linear regression model based on the stimulus presentation timing, which was used to test if the signal during the task periods was statistically different from that of the baseline rest periods. Since data was analyzed as a block-design, missed or incorrect individual trials were not excluded. The details of the analysis are presented in S1 Text (also see [23] for review). In brief, we used an autoregressive-whitened robust regression solution to the regression model as detailed in Barker *et al*. [24]. The significance of changes in brain activity (total-hemoglobin) was tested using a t-test on the regression coefficients for each model. Following analysis of the general linear model for each scan, a mixed effects group level model was used to examine both the average group responses and covariate analysis with age and gait speed. Gait speed was used as a covariate as it has been shown to be a predictor for risk of falls and general health [25, 26]. Subject number and gender were controlled as random variables in the model. All statistical results are shown as a Benjamini-Hochberg false-discovery rate corrected p-value (denoted q-value) [27], accounting for all task comparisons and fNIRS channels. All first and second level statistical analysis was done in Matlab using an open-source custom analysis toolbox written by the authors [23] (see S1 Text). For each estimate, a power analysis was also performed and reported on a per channel and condition basis since in an fNIRS study, the signal-to-noise level of measurements varies depending on the coupling of the fibers and the scalp and according to the presence of hair under the probe. The power of each statistical test was computed based on the probability that the minimum detectable change needed to reject the null hypothesis exceeded the measurement noise (see [28]).

Finally, the fNIRS head cap was registered to a functionally labeled atlas brain to define regions of interest from Brodmann area 45 and 46 (superior frontal cortex) and area 10 (middle frontal cortex) on both hemispheres (see Table 2, S1 Table and S2 Fig). The t-statistic contrast from each region-of-interest was computed from a weighted contribution of each channel and the full (channel by channel) covariance matrix. Further details on the analysis methods used in this study can be found in S1 Text. Based on the registration, the fNIRS probe was located overlying the middle frontal and superior frontal gyrus regions (Fig 1A) based on the statistical parametric mapping (SPM) automatic anatomical labeling (AAL2) database [29].(Table 2).

## Results

### Behavioral performance

Cognitive test performance for the subject cohort, including raw scores and percentiles relative to a normative population, are shown in Table 3. Subjects demonstrated the expected increases in reaction time as the Stroop Task requirements became more complex. The percentiles reveal that the subject sample performed slightly below age-normed values, and that about 5–8 subjects performed well below average, based on a percentile of less than 25.

**Table 3:** Cognitive test performance.

Task	n	Mean (sd) Raw	Mean (sd) Percentile	Number of subjects below 25%
Symbol Digit Coding (SDC) Number Correct	18	28 (12)	53 (33)	5
Stroop (ST-SRT) Simple Reaction Time (ms)	19	466 (180)	46 (29)	5
Stroop (ST-CONG) Congruent Reaction Time (ms)	19	877 (196)	40 (36)	7
Stroop (ST-INCONG) Incongruent Reaction Time (ms)	19	1017 (195)	39 (36)	8
Shifting Attention Test (SAT) Number Correct	17	27 (13)	35 (31)	8
Shifting Attention Test (SAT) Reaction Time (ms)	17	1360 (175)	40 (26)	4

SDC, Symbol Digit Coding; SAT, Shifting Attention Test; ST, Stroop Test; SRT, Simple Reaction Time; CONG, Congruent; INCONG, Incongruent. For outcome measures, a greater number correct, and smaller reaction time indicates better performance. Greater percentile values always indicate better performance

### fNIRS activation

**Fig 2:**
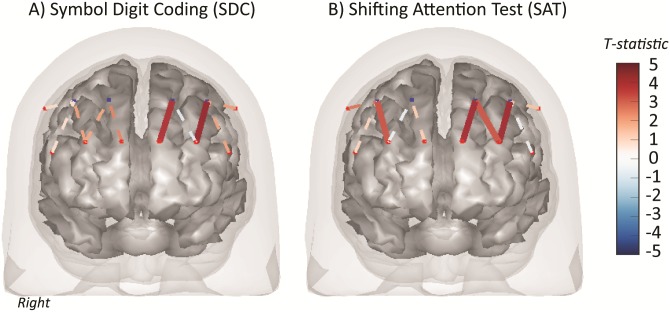
Functional NIRS activation (SDC and SAT). Functional NIRS brain activity maps (total-hemoglobin) for the Symbol Digit Coding test and Shifting Attention Task. The color of the channel/line indicates the T-statistic according to the color bar (right) with solid lines showing channels significant at a false-discovery rate of q<0.05 corrected for all task comparisons.

The fNIRS activation maps for the SDC and SAT tasks relative to the seated rest baseline are shown in Fig 2 (T-scores and q-values listed in Table 4). The SDC task showed increased activation (q<0.05) on the left hemisphere in channels S5-D3 (BA-10) and S6-D4 (BA-45). In region-of-interest analysis, the left BA-10 was significantly activated (T = 2.81; q = 0.042; Table 5) for the SDC task.

**Table 4:** Functional NIRS source-detector channels showing significant increases in total hemoglobin concentration compared with the baseline rest condition.

fNIRS Channel	Nearest ROI		Contrast		β [μM]	StdErr-β	T-statistic	p-value	q-value	power
Source 5: Detector 3	Left Superior Frontal	BA-10	Symbol Digit Coding	SDC	0.67	0.17	3.89	1.1 x 10^−4^	2.2 x 10^−3^	0.57
Source 6: Detector 4	Left Superior Frontal	BA-45	Symbol Digit Coding	SDC	0.43	0.10	4.25	2.4 x 10^−5^	8.6 x 10^−4^	0.57
Source 3: Detector 1	Right Superior Frontal	BA-45	Shifting Attention Task	SAT	0.37	0.12	3.20	1.4 x 10^−3^	1.6 x 10^−2^	0.58
Source 5: Detector 3	Left Superior Frontal	BA-10	Shifting Attention Task	SAT	0.71	0.17	4.08	4.9 x 10^−5^	1.1 x 10^−3^	0.57
Source 6: Detector 3	Left Superior Frontal	BA-10	Shifting Attention Task	SAT	0.25	0.08	3.19	1.5 x 10^−3^	1.6 x 10^−2^	0.59
Source 6: Detector 4	Left Superior Frontal	BA-45	Shifting Attention Task	SAT	0.38	0.09	4.09	4.7 x 10^−5^	1.1 x 10^−3^	0.57
Source 3: Detector 1	Right Superior Frontal	BA-45	Simple Reaction Time	ST-SRT	0.38	0.09	4.13	3.9 x 10^−5^	1.1 x 10^−3^	0.57
Source 1: Detector 1	Right Middle Frontal	BA-46	Simple Reaction Time	ST-SRT	0.28	0.08	3.46	5.6 x 10^−4^	7.6 x 10^−3^	0.58
Source 5: Detector 3	Left Superior Frontal	BA-10	Simple Reaction Time	ST-SRT	0.40	0.13	3.17	1.6 x 10^−3^	1.6 x 10^−2^	0.59
Source 6: Detector 4	Left Superior Frontal	BA-45	Simple Reaction Time	ST-SRT	0.34	0.10	3.45	5.8 x 10^−4^	7.6 x 10^−3^	0.58
Source 3: Detector 1	Right Superior Frontal	BA-45	Stroop Congruent	ST-CONG	0.28	0.07	3.75	1.9 x 10^−4^	3.5 x 10^−3^	0.57
Source 1: Detector 1	Right Middle Frontal	BA-46	Stroop Congruent	ST-CONG	0.21	0.07	2.79	5.4 x 10^−3^	5.0 x 10^−2^	0.59
Source 5: Detector 3	Left Superior Frontal	BA-10	Stroop Congruent	ST-CONG	0.43	0.12	3.70	2.3 x 10^−4^	3.8 x 10^−3^	0.57
Source 6: Detector 4	Left Superior Frontal	BA-45	Stroop Congruent	ST-CONG	0.32	0.09	3.39	7.3 x 10^−4^	8.8 x 10^−3^	0.58
Source 8: Detector 4	Left Middle Frontal	BA-46	Stroop Congruent	ST-CONG	0.16	0.04	3.68	2.5 x 10^−4^	3.8 x 10^−3^	0.57
Source 3: Detector 2	Right Superior Frontal	BA-10	Stroop Incongruent	ST-INCONG	0.39	0.09	4.59	5.1 x 10^−6^	2.3 x 10^−4^	0.56
Source 3: Detector 1	Right Superior Frontal	BA-45	Stroop Incongruent	ST-INCONG	0.47	0.10	4.62	4.5 x 10^−6^	2.3 x 10^−4^	0.56
Source 1: Detector 1	Right Middle Frontal	BA-46	Stroop Incongruent	ST-INCONG	0.44	0.09	4.99	7.3 x 10^−7^	1.3 x 10^−4^	0.56
Source 5: Detector 3	Left Superior Frontal	BA-10	Stroop Incongruent	ST-INCONG	0.53	0.12	4.61	4.7 x 10^−6^	2.3 x 10^−4^	0.56
Source 3: Detector 1	Right Superior Frontal	BA-45	Stroop Incongruent: Age	ST-INCONG:Age	0.61	0.21	2.83	4.7 x 10^−3^	4.5 x 10^−2^	0.59

BA, Brodmann Area; SDC, Symbol Digit Coding; SAT, Shifting Attention Test; ST, Stroop Test, SRT, Simple Reaction Time; CONG,. Congruent; INCONG, Incongruent. Only channels meeting false-discovery corrections of q<0.05 are shown. The type-II error power (1-β) is given which was computed for each channel and statistical test.

**Table 5:** Regions-of-interest (ROI) with significant increases in total hemoglobin concentration compared with the baseline rest condition.

ROI		Contrast		β [μM]	StdErr-β	T-statistic	p-value	q-value	power
BA-10	Right	Symbol Digit Coding	SDC	0.76	0.26	2.94	3.32 x 10^−3^	1.86 x 10^−2^	0.59
BA-45	Left	Symbol Digit Coding	SDC	0.69	0.23	2.97	3.06 x 10^−3^	1.86 x 10^−2^	0.59
BA-46	Left	Symbol Digit Coding	SDC	0.74	0.24	3.01	2.64 x 10^−3^	1.84 x 10^−2^	0.59
BA-10	Left	Shifting Attention Task	SAT	0.96	0.34	2.83	4.74 x 10^−3^	1.89 x 10^−2^	0.59
BA-46	Left	Shifting Attention Task	SAT	1.06	0.31	3.42	6.54 x 10^−4^	6.09 x 10^−3^	0.58
BA-10	Left	Simple Reaction Time	ST-SRT	0.72	0.27	2.64	8.50 x 10^−3^	3.24 x 10^−2^	0.60
BA-45	Left	Simple Reaction Time	ST-SRT	0.68	0.24	2.85	4.43 x 10^−3^	1.89 x 10^−2^	0.59
BA-46	Left	Simple Reaction Time	ST-SRT	0.93	0.26	3.55	3.99 x 10^−4^	4.77 x 10^−3^	0.57
BA-45	Right	Simple Reaction Time	ST-SRT	0.90	0.25	3.59	3.39 x 10^−4^	4.77 x 10^−3^	0.57
BA-46	Right	Simple Reaction Time	ST-SRT	0.85	0.24	3.58	3.65 x 10^−4^	4.77 x 10^−3^	0.57
BA-45	Left	Simple Reaction Time * Gait	ST-SRT:Gait	2.29	0.79	2.91	3.70 x 10^−3^	1.86 x 10^−2^	0.59
BA-46	Left	Simple Reaction Time * Gait	ST-SRT:Gait	2.37	0.82	2.88	4.00 x 10^−3^	1.86 x 10^−2^	0.59
BA-10	Left	Stroop Congruent	ST-CONG	0.69	0.27	2.55	1.09 x 10^−2^	3.96 x 10^−2^	0.60
BA-45	Left	Stroop Congruent	ST-CONG	0.84	0.26	3.24	1.24 x 10^−3^	9.48 x 10^−3^	0.58
BA-46	Left	Stroop Congruent	ST-CONG	0.82	0.24	3.45	5.76 x 10^−4^	6.03 x 10^−3^	0.58
BA-10	Right	Stroop Congruent	ST-CONG	0.63	0.22	2.90	3.80 x 10^−3^	1.86 x 10^−2^	0.59
BA-45	Right	Stroop Congruent	ST-CONG	0.83	0.25	3.36	7.92 x 10^−4^	6.64 x 10^−3^	0.58
BA-46	Right	Stroop Congruent	ST-CONG	0.94	0.24	3.95	8.30 x 10–5	2.48 x 10^−3^	0.57
BA-10	Left	Stroop Incongruent	ST-INCONG	0.78	0.28	2.84	4.61 x 10^−3^	1.89 x 10^−2^	0.59
BA-10	Right	Stroop Incongruent	ST-INCONG	1.05	0.27	3.93	8.87 x 10–5	2.48 x 10^−3^	0.57
BA-45	Right	Stroop Incongruent	ST-INCONG	0.79	0.22	3.58	3.59 x 10^−4^	4.77 x 10^−3^	0.57
									

BA, Brodmann Area; SDC, Symbol Digit Coding; SAT, Shifting Attention Test; ST, Stroop Test, SRT, Simple Reaction Time; CONG,. Congruent; INCONG, Incongruent. Regions-of-interest analysis (see S1 Text, S1 Fig) showing significant activations at a false-discovery rate of q<0.05. The left/right Brodmann areas (BA-) 10, 45, and 46 were examined as the six regions in the analysis. Only regions showing statistical changes are reported. The type-II error power (1-β) is given which was computed for each channel and statistical test.

In contrast, the SAT showed bilateral activity (Fig 2). Increases in activation were observed for channels S5-D3 (left BA-10), S6-D3 (left BA-10), and S6-D4 (left BA-45) on the left side. Channel S3-D1 (right BA-45) on the right hemisphere was also activated. In region-of-interest analysis, only the left BA-10 region met statistical significance (T = 3.09; q = 0.035; Table 5).

**Fig 3:**
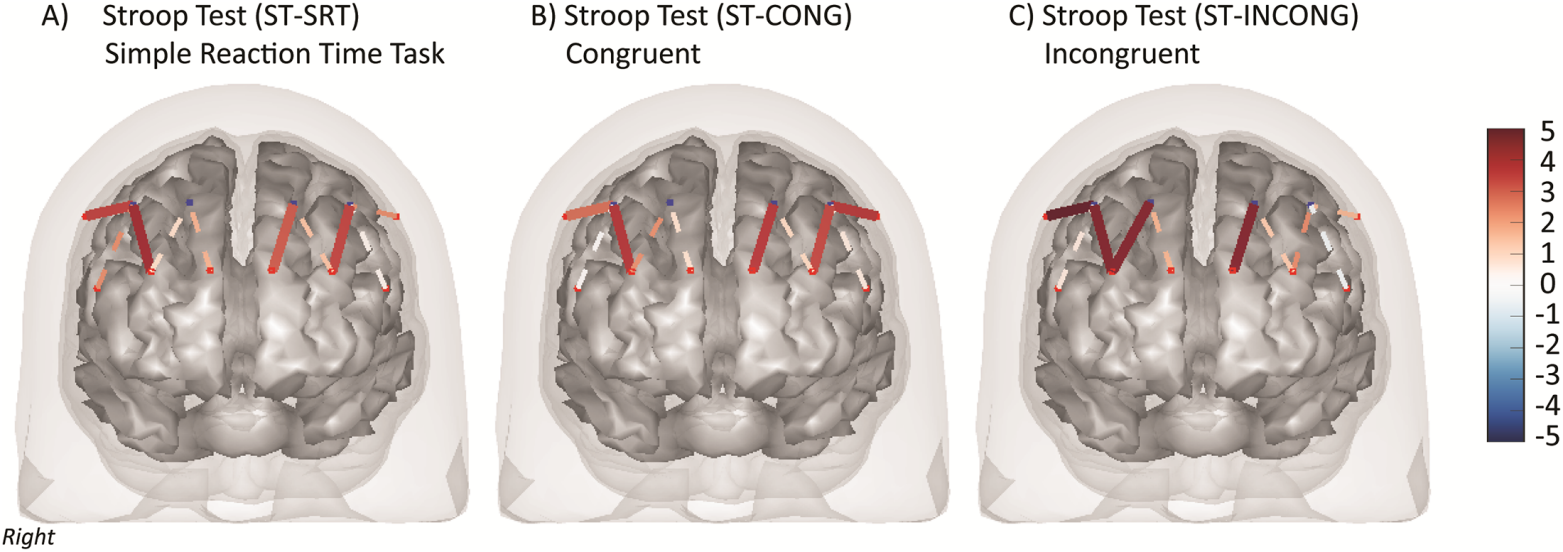
Functional NIRS activation (STROOP tasks). FNIRS brain activity maps (total-hemoglobin) for the A) Simple reaction time task (ST-SRT), B) congruent Stroop (ST-CONG) and C) incongruent Stroop (ST-INCONG) tasks. The color of the channel/line indicates the T-statistic according to the color bar (right) with solid lines showing channels significant at a false-discovery rate of q<0.05 corrected for all task comparisons.

Fig 3 shows the fNIRS brain activity maps for the three variations of the Stroop task. The values of the effects for all significant channels (q<0.05) are provided in Table 4. Bilateral increased activation of channels S1-D1 (right BA-46), S3-D1 (right BA-45) and S5-D3 (left BA-10) occurred in all three Stroop tasks. Channel S6-D4 (left BA-45) was more active in the Stroop simple reaction time task and congruent task compared to baseline, but not the incongruent task. Channel S8-D4 (Left BA-46) was only active during the Stroop congruent task, and channel S3-D2 (Right BA-10) was only active in the incongruent task.

**Fig 4:**
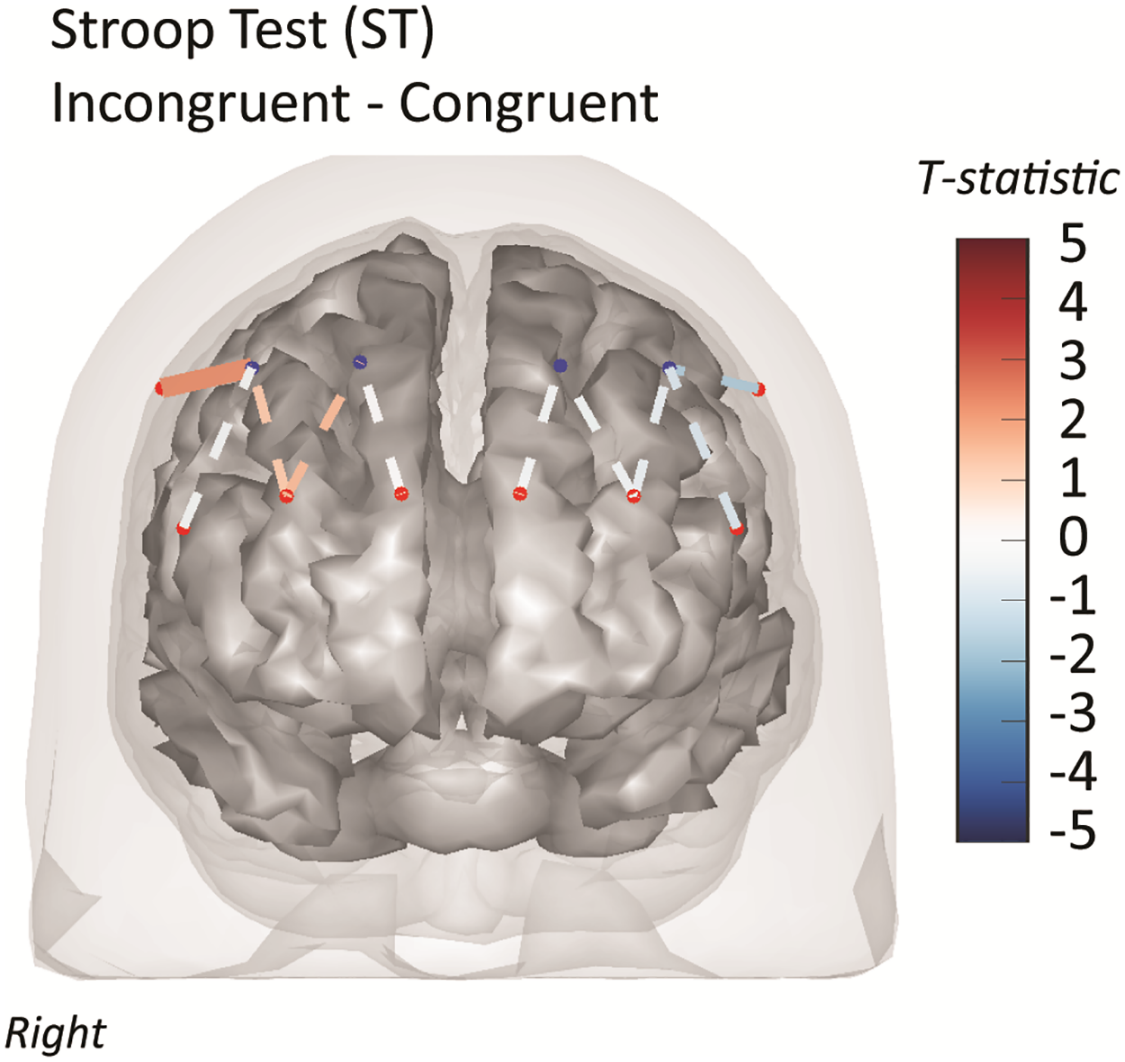
Functional NIRS activation (STROOP Incongruent verses Congruent). Functional NIRS brain activity maps (total-hemoglobin) for the incongruent Stroop (ST-INCONG) versus the congruent Stroop (ST-CONG) tasks. The color of the channel/line indicates the T-statistic according to the color bar (right) with solid lines showing channels significant at a false-discovery rate of q<0.05 corrected for all task comparisons and fNIRS channels examined in this work.

The regions of interest analysis demonstrated that the left BA-10 and right BA-45/46 was activated in all conditions of the Stroop task (Table 5). The left BA-10 was active in the Stroop simple, congruent and incongruent tasks, while the left BA-45 and left BA-46 were active in the simple and congruent tasks. Fig 4 shows the activation map of the Stroop Incongruent versus Stroop Congruent task as described in the main text. Greater total-hemoglobin activation was observed in the right middle frontal region (BA-46).

**Fig 5:**
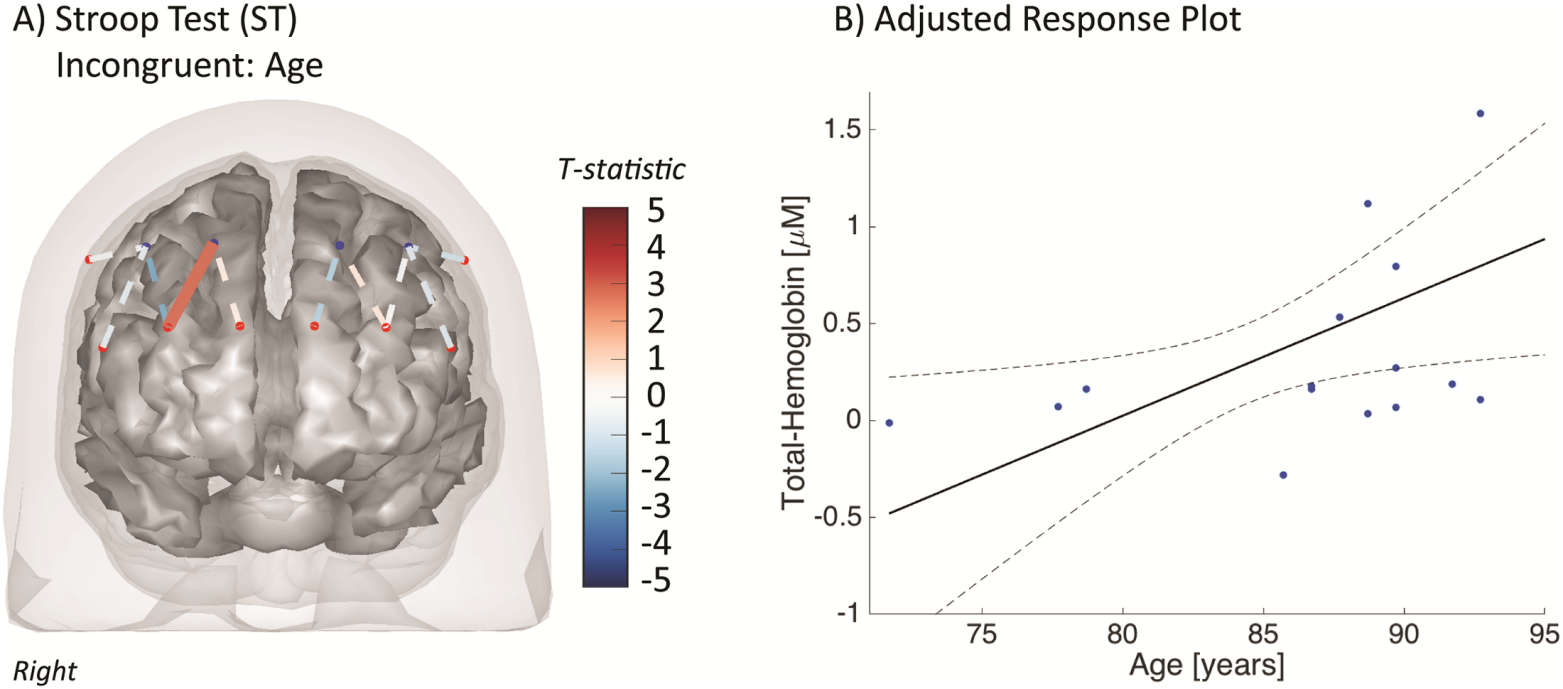
Age-related functional NIRS activation (STROOP-AGE). The activation of the incongruent Stroop test (ST-INCONG) was positively associated with participant age in the right superior-frontal region) in the mixed effects model (see S1 Text). The panel (right) shows a scatter plot of the weighted adjusted beta’s from the first-level (single subject) general linear models versus the participant age.

The correlation of brain activity with both age and gait speed was also examined using the group-level mixed effects model (see S1 Text). The average gait speed for the population was 0.74 m/s (SD 0.26 m/s; range = [0.38–1.20m/s]). No brain regions or tasks were found to correlate with the gait speed. For age, we found that only in channel S3-D2 (right superior frontal; BA-10) the ST-INCONG was positively associated with age (T = 2.83; q = 0.045; shown in Fig 5). This region-of-interest was correlated (T = 2.13; p = 0.03; q = 0.17) for this same task and also for the ST-CONG task (T = 1.93; p = 0.05; q = 0.23). There was a positive association in this region with the SDC (T = 1.85; p = 0.06; q = 0.24) and negatively with the SAT (T = -1.78; p = 0.064; q = 0.25). None of these regions passed false discovery corrections.

## Discussion

The primary findings of this study indicated increased activation above baseline for channels overlying the left BA-10 region-of-interest (ROI) during the Symbol Digit Coding test and Shifting Attention Test. During all versions of the Stroop test, increased activity was observed in the channels above the right BA-10 and BA-45 and left BA-10 ROIs.

### Behavioral performance

With mean values within 5% of age-normed reference values, the group performance on the SDC and Stroop SRT reflected that the cognitive processing time and visuomotor reaction times of this sample to be representative of the general population. However, the sample lagged behind their age cohort in performance of the Stroop congruent and incongruent tests and the SAT, demonstrated by the lower mean values (40th, 39th, and 35th percentile, respectively) relative to their peers. Furthermore, a larger percentage of subjects had scores below the 25th percentile. These tests assess additional executive functions including sustained and selective attention, inhibition, and set shifting. The relatively lower performance of this group, compared with peer-performance, probably reflects the greater prevalence of cognitive impairment in residents of residential care communities compared with older adults living in the community [2], and may portend a need for greater assistance with activities that require these complex executive functions.

Assessment of cognitive function in older adults using computer-based assessment has been investigated previously. The American Psychological Association acknowledged the potential benefit of administering technology based assessments for individuals of limited mobility, while recognizing potential limitations for older adults who do not routinely use computers [30]. Espeland [4] reported higher rates of incomplete data as subjects became older, or had less experience with computers. Therefore, it is possible that some residents of RCCs may not be able to perform a computer-based assessment if they have some cognitive impairment. In our experience, only a few of the subjects had initial difficulty performing the test on the laptop. This low rate of difficulty may have been assisted by use of a custom keyboard with color-coded keys provided by the test manufacturer.

### fNIRS activation

The primary function attributed to the SDC test is processing speed, but it also draws upon visual processing abilities and presumably some level of sustained attention must be used to complete the task over 90sec. Of the three tests, the SDC test elicited increased activation in the least amount of channels, and only in the left hemisphere (specifically left BA-10 and BA-45). In contrast, a synthesis of the neuroimaging literature reported that, in young adults, the right BA-10 and BA-46 Brodmann areas are active during sustained attention tasks and that the right BA-10 is active during visual perception of objects [31]. However, older adults more frequently display more diffuse activation, which could explain the left-sided activation in this study [6]. Additionally, Rosano et al. [9] previously discovered an association between greater activation in the left dorsolateral prefrontal cortex and improved digit symbol substitution test performance in active older adults compared with sedentary older adults.

The SAT elicited activity in similar regions and further recruited a channel over the right BA-45. However, the left BA-10 was the only region to survive the region of interest analysis. The primary ability tested with the SAT is set shifting, or the ability to alter responses based on changing rules. Other common ways to assess this function include the Trail Making B Test or the Wisconsin Card Sorting Test. A review of functional imaging studies examining activation during the Wisconsin Card Sorting Test in young adults has demonstrated predominantly left hemisphere activation in BA-45, right activation in BA-10, and bilateral activation in BA-46 [32–34]. During both written and computerized versions of the Trail Making Test, increased activity has been documented in the prefrontal cortex bilaterally using fNIRS [12, 15], to a greater degree anteriorly than laterally [12]. Consequently, our findings in older adults residing in residential care communities generally replicate the results observed in young adults, although one may have expected stronger bilateral activation in the current study.

The ST had the greatest frequency of channels that produced significantly increased activation. Our findings of increased activation bilaterally in BA-10 during the simple, congruent and incongruent test components and greater activation in right BA-45 corroborate numerous previous findings of increased activity in the inferior and superior frontal gyri [7, 8, 11, 14]. We specifically found that the right BA-10 is significantly activated more during the incongruent compared to the congruent condition. Several reports have suggested that aging plays a role in how the brain performs the task. We found that the right BA-45 was associated with age. For instance, several studies have documented increased activation in older adults compared with young adults during Stroop performance [7, 35], consistent with the generalized increased activation theories [6]. An important contribution of future work will be the investigation of how performance during the Stroop test is related to brain activation in a larger sample of older adults, some of who demonstrate mild cognitive impairment.

The findings of increased total hemoglobin concentration during cognitive test performance build upon previous fNIRS investigations, particularly with respect to tasks that increase working memory. In these studies, older and young adults have demonstrated greater oxyhemoglobin concentration with greater working memory load, and greater bilateral activation in older adults [36]. In addition, a larger decline in performance as the working memory load increased resulted in greater bilateral activation [37]. However, individuals with mild cognitive impairment may produce decreased oxyhemoglobin concentrations. [38, 39] Thus it appears that using portable cognitive testing and neuroimaging methods such as fNIRS may help to document evolving cognitive impairments in at-risk populations living in the community.

### Limitations of this study

One of the limitations of fNIRS is that it only measures functional changes in brain activity and does not provide any structural information about the brain. In this study, we used the Colin27 atlas for registration and the definition of regions-of-interest. Because fNIRS measures brain signals by sending light in from the surface of the scalp, these signals are sensitive to changes and variability in the cortical depth of the brain region from the scalp as well as extra-cerebral factors like skin, fat, skull, and cerebral spinal fluid thicknesses. In particular, in an aging population, brain atrophy will cause this cortical depth to increase, thereby lowering the sensitivity of the fNIRS to this brain region and resulting in age-related decreases in the reported brain activity by fNIRS. In our results, we saw an increase in the brain activity for the incongruent Stroop task with increasing age; however, this effect could be underestimated if there was additional atrophy affecting these measurements. The potential for systematic biases based on atrophy or other anatomical differences between populations must be considered further when looking at population differences (e.g. brain activity of younger versus older subject groups). Further, fNIRS is limited to cortical measurements within the regions that are covered by the probe used (bilateral BA-10, 45, and 46 were covered). Therefore, no information about brain activity in regions outside of this probe volume is available and we cannot comment on the possibility of recruitment of other brain regions, which were not measured in this age group. Based on analysis of this data, we found most of our statistical tests to be powered around 60% with N = 19 participants in this pilot study. In future studies, to obtain 80% power, a sample size of at least 32 participants would be needed (or more if additional covariates are considered), which we view as reasonable for recruitment purposes.

As a further limitation, the measurements made here were performed using an 808nm source and thus measure total hemoglobin compared to the standard in fNIRS, which measures both oxy- and deoxy-hemoglobin. In this study, the 808 wavelength had been used in order to double the number of source positions available using our TechEN NIRS-2 system, which is limited to a total of four laser diode slots. By using a 4x1 [wavelength] setup instead of the usual 2x2, we were able to measure more brain regions. In future work, newly availability portable systems with a larger number of light sources and detectors could be used to further extend these measurements as well as offer the ability to measure both oxy- and deoxy-hemologbin.

### Future directions

This is one of the first studies to use portable fNIRS brain imaging technology to look at brain activity signals in an older population living in residential care communities. Specifically, this work demonstrates the utility of fNIRS to access this under-studied population using instruments that were brought directly into the resident’s home. The current study used a relatively small set of channels, thus limiting our ability to determine if the changes in brain activation were specific to the frontal region covered by our probe, or was more widespread. In the future, higher-density optode arrays with greater coverage will allow us to more precisely locate the changes in brain activation during these tasks. As the fNIRS technology becomes more widely available and portable, and if longitudinal studies determine that the outcomes have some ability for predicting future changes in health status, the findings may hold promise for managing care of the residents. Our results suggest that using fNIRS during performance of a computerized Stroop test would elicit the greatest activation, and be a starting point for examining changes in status. However, it would also be important to relate the activation changes to test performance, which we were not able to do in this study.

## Supporting information

S1 Fig Schematic of fNIRS measurement configuration. Schematic of the fiber bifurcations used to sample eight total source positions on the head cap using only the four lasers available on the NIRS instrument. The fNIRS data was collected using a TechEn NIRS-2 system, which has a total of 4 lasers (customized to all be at 808nm) and 4 detector positions. We used bifurcated fiber optics on the lasers to send each of these four lasers to two separate positions on the head, thereby doubling the number of concurrent measurements. The same laser was sent to opposite hemispheres and a staggered positioning to ensure that the light from each position could be uniquely identified.
(DOCX)

S2 Fig Location of brain regions-of-interest. The fNIRS probe was registered to the Colin27 atlas, which was used in combination with the automatic anatomical labeling toolbox (aal2) to label the Brodmann areas 10, 45, and 46. The images above show topology maps (Clarke azimuthal map projection) showing the depth of the nearest cortical point in the region-of-interest to the surface of the head. A yellow indicates a depth of greater then 30mm, which would be inaccessible to fNIRS.
(DOCX)

S1 Table Spatial weights of regions-of-interest. Contrast weights used for definition of the six regions-of-interest based on the relative sensitivity of each channel to the region derived from the optical forward model.
(DOCX)

S1 Text Analysis of NIRS data. (DOCX)
