# Supplementary material for: Functional imaging of cognition in an old-old population: A case for portable functional near-infrared spectroscopy
Source: PLoS One. 2017 Oct 12;12(10):e0184918. doi: 10.1371/journal.pone.0184918 (PMC5638236; doi:10.1371/journal.pone.0184918)
Supplement: S1 Fig — Schematic of the fiber bifurcations used to sample eight total source positions on the head cap using only the four lasers available on the NIRS instrument. The fNIRS data was collected using a TechEn NIRS-2 system, which has a total of 4 lasers (customized to all be at 808nm) and 4 detector positions. We used bifurcated fiber optics on the lasers to send each of these four lasers to two separate positions on the head, thereby doubling the number of concurrent measurements. The same laser was sent to opposite hemispheres and a staggered positioning to ensure that the light from each position could be uniquely identified. (DOCX) [file pone.0184918.s001.docx]

**S1 Fig. Schematic of fNIRS measurement configuration**


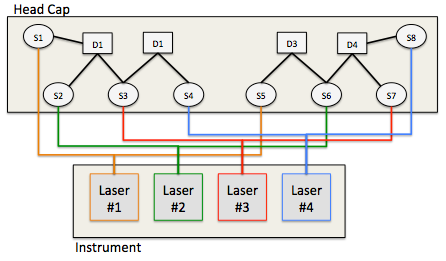


Schematic of the fiber bifurcations used to sample eight total source positions on the head cap using only the four lasers available on the NIRS instrument. The fNIRS data was collected using a TechEn NIRS-2 system, which has a total of 4 lasers (customized to all be at 808nm) and 4 detector positions. We used bifurcated fiber optics on the lasers to send each of these four lasers to two separate positions on the head, thereby doubling the number of concurrent measurements. The same laser was sent to opposite hemispheres and a staggered positioning to ensure that the light from each position could be uniquely identified.
