# Supplementary material for: Functional imaging of cognition in an old-old population: A case for portable functional near-infrared spectroscopy
Source: PLoS One. 2017 Oct 12;12(10):e0184918. doi: 10.1371/journal.pone.0184918 (PMC5638236; doi:10.1371/journal.pone.0184918)
Supplement: S2 Fig — The fNIRS probe was registered to the Colin27 atlas, which was used in combination with the automatic anatomical labeling toolbox (aal2) to label the Brodmann areas 10, 45, and 46. The images above show topology maps (Clarke azimuthal map projection) showing the depth of the nearest cortical point in the region-of-interest to the surface of the head. A yellow indicates a depth of greater then 30mm, which would be inaccessible to fNIRS. (DOCX) [file pone.0184918.s002.docx]

**S2 Figure. Location of brain regions-of-interest**


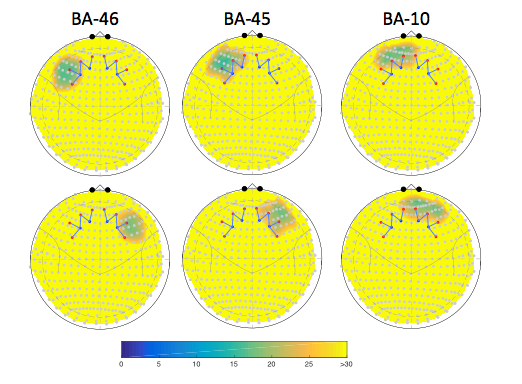


The fNIRS probe was registered to the Colin27 atlas, which was used in combination with the automatic anatomical labeling toolbox (aal2) to label the Brodmann areas 10, 45, and 46. The images above show topology maps (Clarke azimuthal map projection) showing the depth of the nearest cortical point in the region-of-interest to the surface of the head. A yellow indicates a depth of greater then 30mm, which would be inaccessible to fNIRS.
