# Supplementary material for: Functional imaging of cognition in an old-old population: A case for portable functional near-infrared spectroscopy
Source: PLoS One. 2017 Oct 12;12(10):e0184918. doi: 10.1371/journal.pone.0184918 (PMC5638236; doi:10.1371/journal.pone.0184918)
Supplement: S1 Table — Contrast weights used for definition of the six regions-of-interest based on the relative sensitivity of each channel to the region derived from the optical forward model. (DOCX) [file pone.0184918.s003.docx]

**S1 Table. Spatial weights of regions-of-interest**

|  | **BA-10 (Left)** | **BA-10 (Right)** | | **BA-45 (Left)** | | **BA-45 (Right)** | **BA-46 (Left)** | **BA-46 (Right)** |
| --- | --- | --- | --- | --- | --- | --- | --- | --- |
| Source 1 : Detector 1 | 0.00% | 0.22% | | 0.00% | | 27.66% | 0.00% | 12.73% |
| Source 2 : Detector 1 | 0.01% | 3.14% | | 0.00% | | 52.80% | 0.00% | 29.32% |
| Source 3 : Detector 1 | 0.11% | 12.73% | | 0.00% | | 16.64% | 0.00% | 28.34% |
| Source 3 : Detector 2 | 0.94% | 36.34% | | 0.00% | | 2.60% | 0.02% | 20.84% |
| Source 4 : Detector 2 | 6.06% | 41.52% | | 0.00% | | 0.31% | 0.13% | 8.70% |
|  | | | *midline* | |  | | | |
| Source 5 : Detector 3 | 48.45% | 5.14% | | 0.36% | | 0.00% | 15.35% | 0.06% |
| Source 6 : Detector 3 | 30.32% | 0.80% | | 3.32% | | 0.00% | 23.61% | 0.01% |
| Source 6 : Detector 4 | 11.35% | 0.10% | | 23.55% | | 0.00% | 29.68% | 0.00% |
| Source 7 : Detector 4 | 2.65% | 0.01% | | 42.21% | | 0.00% | 20.32% | 0.00% |
| Source 8 : Detector 4 | 0.10% | 0.00% | | 30.56% | | 0.00% | 10.89% | 0.00% |

Contrast weights used for definition of the six regions-of-interest based on the relative sensitivity of each channel to the region derived from the optical forward model.
