# Supplementary material for: Functional imaging of cognition in an old-old population: A case for portable functional near-infrared spectroscopy
Source: PLoS One. 2017 Oct 12;12(10):e0184918. doi: 10.1371/journal.pone.0184918 (PMC5638236; doi:10.1371/journal.pone.0184918)
Supplement: S1 Text — (DOCX) [file pone.0184918.s004.docx]

**S1 Text: Analysis of NIRS data.**

**Preprocessing.**

FNIRS data is recorded as changes in the light from a source position incident on a detector position (e.g. transmitted between a source-detector pair) as a function of time. These signals are first converted to changes in optical density (optical absorption) over time as given by

$\Delta OD\left( t \right)= -log(I(t)/I_{0})$ Eq. (1)

where I(t) is the intensity of the signal recorded and I_0_ is the reference signal intensity at baseline (taken as the mean DC signal). Since our system only recorded at 808nm, which was done to allow more spatial coverage of the probe using the small instrument, optical density changes are directly proportional to changes in total hemoglobin. 808nm is an isobestic point of hemoglobin, where both oxy- and deoxygenated forms have similar optical absorption.

**Statistical analysis.**

The fNIRS signals (total-hemoglobin changes) between each source-to-detector pair were analyzed using a general linear regression model to test for statistical differences between the baseline and task conditions for each scan. This regression model is described by the equation

$\Delta H=\boldsymbol{X}*\beta+\epsilon$ Eq. (2)

where **X** is the design matrix encoding the timing of stimulus events and *β* is the coefficient (weight) of that stimulus condition for that source-detector channel. *∆H* is the vector of measurements of hemoglobin, which in our case is proportional to optical density changes (Eq. 1). Note that this proportionality constant will cancel in the case of examining t-statistics, so was just taken as unity in this work. For each scan, which consisted of a rest period and a single task condition, the matrix **X** consists of two columns. The first column consists of the task-related regressor and is zero during the rest periods and ones during the task. This is then convolved with an expected canonical hemodynamic response function (HRF) model. The default timing for the HRF model from the program AFNI (1) was used, which is the fairly ubiquitous standard in functional magnetic resonance imaging. Because the task has a long duration (self-paced; average 82s), the choice of HRF function is expected to have almost no effect on the results. It should be noted as detailed in Huppert 2016 (2), the use of the canonical HRF model is mathematically similar to solving the full deconvolution model (e.g. estimating the full shape of the response curve from the data) followed by computing the average t-statistic contrast over a weighted window of time. In particular, these two approaches produce the same t-statistical estimate in the asymptotic limit when the weighted window of time is equal to the normalized temporal shape of the canonical model. Furthermore for independently and identically distributed (i.i.d.) noise, mis-specification of the canonical model results in the introduction of type-II, but not type-I errors, and is equivalent to computing the contrast from the deconvolution model over a non-optimal window of time (2). The second column of the **X** matrix encodes the mean of the rest period and is a column of all ones. Although a common practice is often to include additional columns of discrete cosine transform terms or polynomial trend regressors, this is not necessary using our general linear model formulation (described in the next section) and only the two columns were used in this model. In this study, **X** coded the canonical response to the stimulus condition, but in general, the mathematics in the next section applies to any similar formulation of the model including finite-impulse response (deconvolution) models of the hemodynamic response.

**General linear model.**

The importance of a general linear model in the context of fNIRS analysis is described in (2). A generalization of a linear model refers to the steps taken to relax the statistical assumptions on standard linear regression in order to avoid uncontrolled false-discovery and bias in the estimates. In particular, the two main characteristics of noise in fNIRS data are serially correlated errors due to the slowness of the hemodynamic response and systemic physiology and heavy-tailed noise variations due to motion-related artifacts that are often several fold larger in magnitude then other noise fluctuations. Ordinary linear regression assumes uncorrelated, white, independent noise and so generalizations of the linear model are required in the presence of the non-idealized fNIRS noise. In particular, autoregressive whitening and iterative robust regression methods are used to address these two types of deviations from ideal noise. For a fuller discussion of these principals, see (2).

In order to solve the linear regression model described in Eq. 2, we used an iteratively whitened weighted least-squares (AR-iWLS) model as described in Barker et al. 2013 (3). This regression model uses an n^th^ order auto-regressive (AR) filter (W_AR_) determined by an Akaike model-order (AIC) selection to whiten both sides of this expression, e.g.

$\boldsymbol{W}_{\boldsymbol{AR}}*\Delta H=\boldsymbol{W}_{\boldsymbol{AR}}*\boldsymbol{X}*\beta+\boldsymbol{W}_{\boldsymbol{AR}}*\varepsilon$. Eq. (3)

As described in Barker et al. 2013 (3), the regression model is first solved using robust regression and the residual noise is then fit to an AR model. This filter (W_AR_ ) is applied to both sides of the original model and then resolved and repeated until convergence. This AR filter removes serially correlated errors in the data that result from physiological noise and/or motion artifacts. AR whitening, however, does not address the heavy-tailed noise from motion artifacts. To do this, the AR-whitened model is solved using robust weighted regression, which is a procedure to iteratively down-weight outliers such as motion artifacts.

$\boldsymbol{S\cdot}\boldsymbol{W}_{\boldsymbol{AR}}*\Delta H=\boldsymbol{S\cdot W}_{\boldsymbol{AR}}*\boldsymbol{X}*\beta+\boldsymbol{S\cdot}\boldsymbol{W}_{\boldsymbol{AR}}*\varepsilon$. Eq. (4)

where **S** is

$\boldsymbol{S}\left( \frac{r_{W}}{\sigma} \right)=\left\{ \begin{matrix} 1-\left( \frac{r_{W}}{\sigma\cdot\kappa} \right)^{2} & \left| \frac{r_{W}}{\sigma} \right|<\kappa\\ 0 & \left| \frac{r_{w}}{\sigma} \right|\geq\kappa\end{matrix} \right.$ Eq. (5)

which is simply the square root of Tukey's bisquare function (4) and is the same model as used in Eq. 4 from Barker et al. (3). The tuning constant κ is typically set to 4.685 which provides 95% efficiency of the model in the presence of normally distributed errors (4) *σ_{t}_* is the standard deviation of the residual noise in the model.

Using this model, the regression coefficients (*β*) and their error-covariance is estimated, which is used to define statistical tests between task conditions or baseline. The regression model is solved sequentially for each data file for each subject. All source-detector pairs within a file are solved concurrently yielding a full covariance model of the noise, which is used in group-level and region-of-interest analysis. The estimate of *β* and its covariance matrix is given by the expressions

$\boldsymbol{\beta}=\left( \boldsymbol{X}^{T}\cdot\boldsymbol{W}_{AR}^{T}\cdot{\boldsymbol{S}^{\boldsymbol{T}}\boldsymbol{\cdot S\cdot W}}_{AR}\cdot\boldsymbol{X} \right)^{-1}\cdot\boldsymbol{X}^{T}\cdot\boldsymbol{W}_{AR}^{T}\cdot{\boldsymbol{S}^{\boldsymbol{T}}\boldsymbol{\cdot S\cdot W}}_{AR}\cdot\Delta H$ Eq. (6)

$$\boldsymbol{Cov}_{\boldsymbol{\beta}}=\left( \left( \boldsymbol{W}_{\boldsymbol{AR}}\cdot\boldsymbol{X} \right)^{T}\boldsymbol{*W}_{\boldsymbol{AR}}\cdot\boldsymbol{X} \right)^{-1}\cdot\sigma^{2}$$

$$\sigma^{2}=\left( \boldsymbol{W}_{\boldsymbol{AR}}\cdot\Delta H-\boldsymbol{W}_{\boldsymbol{AR}}\cdot\boldsymbol{X}*\beta\right)^{\boldsymbol{T}}\cdot\left( \boldsymbol{W}_{\boldsymbol{AR}}\cdot\Delta H-\boldsymbol{W}_{\boldsymbol{AR}}\cdot\boldsymbol{X}\cdot\beta\right)$$

The t-statistic for comparing conditions can then be obtained from the expression

$T=c\cdot\beta/\sqrt{c\cdot{Cov}_{\beta}\cdot c^{T}}$ Eq. (7)

where *c* is the contrast vector. For example, if there were five total task conditions, then the test of condition #1 > condition #2 would be given by c = [1 -1 0 0 0]^T^.

**Group level analysis.**

A mixed effects group level model was used for secondary analysis. The data file-level statistical model (*β* and covariance) was used as input with the subject ID and subject gender included as random-effects terms in the model. Age and gait-speed were used as additional covariates in the mixed effects model. The group-level mixed effects model is described by the equation

$\beta=\boldsymbol{A}\cdot\Gamma+\boldsymbol{B}\cdot\Theta+\varepsilon$ Eq. (8)

where *β* is the vector of weights obtained from the linear regression model from Eqs. 2-6) which includes entries for each subject, task condition, and source-detector pair. **A** is the fixed effects model and **B** is the random effects model matrices. An example of the group-level model demonstrating the inclusion of the age variable as a cofactor is given by

$\left[ \begin{matrix} \begin{matrix} \beta_{{Subj}_{A},ST} \\ \beta_{{Subj}_{A},SDC} \\ \beta_{{Subj}_{A},SAT} \end{matrix} \\ \vdots\\ \begin{matrix} \beta_{{Subj}_{N},ST} \\ \beta_{{Subj}_{N},SDC} \\ \beta_{{Subj}_{N},SAT} \end{matrix} \end{matrix} \right]=\left[ \begin{matrix} \begin{matrix} \begin{matrix} 1 & & \\ & 1 & \\ & & 1 \end{matrix} & \begin{matrix} \begin{matrix} {age}_{A} \\ {age}_{A} \\ {age}_{A} \end{matrix} & \begin{matrix} {age}_{A} & & \\ & {age}_{A} & \\ & & {age}_{A} \end{matrix} \end{matrix} \end{matrix} \\ \begin{matrix} \begin{matrix} \begin{matrix} \ddots& \end{matrix} & & \vdots\end{matrix} & \begin{matrix} & \begin{matrix} & & \ddots\end{matrix} \end{matrix} \end{matrix} \\ \begin{matrix} \begin{matrix} 1 & & \\ & 1 & \\ & & 1 \end{matrix} & \begin{matrix} \begin{matrix} {age}_{N} \\ {age}_{N} \\ {age}_{N} \end{matrix} & \begin{matrix} {age}_{N} & & \\ & {age}_{N} & \\ & & {age}_{N} \end{matrix} \end{matrix} \end{matrix} \end{matrix} \right]\left[ \begin{matrix} \begin{matrix} \Gamma_{ST} \\ \Gamma_{SDC} \\ \Gamma_{SAT} \end{matrix} \\ \begin{matrix} \begin{matrix} \Gamma_{Age} \\ \Gamma_{ST:Age} \end{matrix} \\ \Gamma_{SDC:Age} \\ \Gamma_{SAT:Age} \end{matrix} \end{matrix} \right]+\left[ \begin{matrix} \begin{matrix} 1 & \\ 1 & \\ 1 & \end{matrix} \\ \vdots\\ \begin{matrix} & 1 \\ & 1 \\ & 1 \end{matrix} \end{matrix} \right]*\left[ \begin{matrix} \Theta_{A} \\ \vdots\\ \Theta_{N} \end{matrix} \right]+\upsilon$ Eq. (9)

where the terms *Γ_ST_, Γ_SDC_,* and *Γ_SAT_* denote the main group level effects for the three task conditions, *Γ_AGE_* denotes the main effect for the age covariate, and the terms *Γ_X:Age_* denote the interaction terms between the three conditions and age. The second matrix (**B**) and coefficients (*Θ*) denote the random effects terms (here indicating subject as a random effect). Since the covariance of this model is known from the first level model (equation S6), the mixed effects model is then solved for using weighted least-squares regression where a weighting matrix ($\boldsymbol{\Omega}$) is applied to the left and right hand sides of the expression and given by

$\boldsymbol{\Omega}\cdot\beta=\boldsymbol{\Omega}\cdot\boldsymbol{A}*\Gamma+\boldsymbol{\Omega}\cdot\boldsymbol{B}\cdot\Theta+\boldsymbol{\Omega}\cdot\nu$ Eq. (10)

where the whitening matrix is defined as

$\boldsymbol{\Omega}^{\boldsymbol{T}}*\boldsymbol{\Omega}=\boldsymbol{Cov}_{\beta}^{-1}$ Eq. (11)

and $\boldsymbol{Cov}_{\boldsymbol{\beta}}$ is the noise covariance matrix given in Eq. 6, which was estimated from the temporal general linear model. $\boldsymbol{\Omega}$ is estimated from a singular value decomposition of the symmetric covariance matrix. Note that we have written this expression only for one fNIRS measurement channel for simplicity, but in reality this model also includes all source-to-detector channels simultaneously and is given by the form

***Ω*** *^.^ (****A*** *⊗* ***I****_CHAN_) ^.^ Γ+* ***Ω*** *^.^ (****B*** *⊗* ***I****_CHAN_)^.^ Θ + ν*  Eq. (12)

where **I_CHAN_** is an identity matrix of size number of fNIRS source-to-detector pairs and *⊗* is the Kronecker operator. This allows the preservation of the full channel-by-channel covariance matrix, which is used in the region-of-interest model. A maximum likelihood estimate of this model is obtained to estimate the effects terms *Γ* and the covariance matrix $\boldsymbol{Cov}_{\boldsymbol{\Gamma}}$**.** Group level t-statistics are computed using the same formula given in Eq. 7.

**Region-of-interest estimates.**

The fNIRS probe was positioned on the head according to the 10-20 positioning system with the center of the probe positioned around the point FpZ (center of forehead). Based on this positioning, the fNIRS probe was registered to a spherical 10-20 template, which is part of the SPM8 toolbox (www.fil.ion.ucl.ac.uk/spm/). Based on the registration of the fNIRS probe to the anatomical atlas the expected relative sensitivity of each fNIRS source-to-detector channel can be estimated from the location and depth of anatomically defined regions through the optical forward model (**L**). The optical forward model (see (5)) defines the sensitivity of the measurements in channel space to underlying changes in the brain space. This model is calculated by estimation or simulation of the diffusion of light through the tissue (e.g. (6, 7)). The optical forward model provides an estimate of the expected signal changes for the fNIRS measurement geometry given by the expression

$Y_{source-detector}=\boldsymbol{L}\cdot\mu_{Volume}$ Eq. (13)

where Y_source-detector_ is the measurement for a specific fNIRS probe, **L** is the forward model relating that probe layout and registration to the underlying head/brain, and μ_volume_ is the underlying change in optical absorption in the volume.

Eq. 7 describes the calculation of a t-statistic testing a hypothesis given by the contrast vector (*c*). In other words, the contrast vector is a model of the expected response, which is being statistically compared to the data. For example, in Huppert 2016 (2), we showed that the canonical model in the time-series GLM equation (Eq. 2) is near equivalent to using a full temporal deconvolution model (e.g. FIR based design matrix) followed by computing the t-statistic (via Eq. 7) using a contrast vector that matched the shape of the expected hemodynamic response (e.g. *c* is equal to the normalized assumed canonical time course). This principal also applies in the context of a statistical test of a region-of-interest. Namely, given the estimates of the effects terms *Γ* and the covariance matrix $\boldsymbol{Cov}_{\boldsymbol{\Gamma}}$ for the full fNIRS probe from Eq. 12, the region-of-interest is given by the same form as Eq. 7. Here the contrast vector (*c*) encodes both the task condition and channels (source-to-detectors) being pooled for the statistical test. Just like the time-series model, this contrast vector should encode the hypothesis we wish to test, but in the case of the region-of-interest, the contrast describes the hypothesis about the spatial distribution of activity.

To test for statistical activity from specific anatomically based region-of-interest, we can use the optical forward model and Eq. 13 to define the hypothesis of what the activity pattern in channel-space should look like based on the location in volume (brain) space. In other words, to form the null hypothesis testing for activity in a specific region-of-interest, the contrast vector (c) is given by

$c_{roi}=\boldsymbol{L}\cdot{Mask}_{ROI}$ Eq. (14)

where

${Mask}_{ROI}(r)=\left\{ \begin{matrix} \begin{matrix} 1 \\ 0 \end{matrix} & \begin{matrix} if r \in ROI \\ else \end{matrix} \end{matrix} \right.$ Eq. (15)

The t-test for a specific region-of-interest is then given by

$T_{ROI}=c\cdot\Gamma/\sqrt{c*{Cov}_{\Gamma}*c^{T}}$ Eq. (16)

and

$c = c_{COND} \cdot c_{ROI}$ Eq. (17)

where C_ROI_ and C_COND_ are the contrast vectors for the region-of-interest and for the pooling of conditions as previously given in Eq. 7. It should be noted that Eq. 17 tests the null hypothesis that the signal from the region-of-interest is equal to zero. This does not test if the activity specifically came from only that region. In other words, this doesn’t test if the entire region is active or just a subset. It also doesn’t rule out that the activity could have been from a nearby region, which was also covered by that source-detector pair. This also assumes the optical forward model and probe registration are accurate. Any mismatch in the registration or forward model (e.g. as a result of anatomical differences including brain atrophy) will mean that the contrast vector is testing a slightly non-optimal hypothesis. As we noted in discussion of the temporal linear model (Eq. 2) and the temporal contrast vector (c_COND_), using a sub-optimal contrast vector for the hypothesis is equivalent to using the wrong time-window for computing the effect. In this case, it is akin to a weighted average across the wrong combination of channels. This introduces type-II error (e.g. the false-negative rate will increase and one might miss activity that actually was significant), but doesn’t introduce type I error (false-positive rate).

In this study, we used six regions-of-interest from Brodmann areas 10, 45, and 46 on the left and right hemispheres. Given the registration of the fNIRS probe relative to the Colin27 atlas (8), the location of these regions is shown in S2 Fig. The automatic anatomical labeling toolbox (aal2; (9)) was used to define the anatomical and Brodmann areas.

Based on the registration of the fNIRS probe, the optical forward model was estimated at 808nm based on a single-layer (homogeneous) head model derived from the Colin27 atlas with optical properties at 808nm [µs’= 1.12 mm^-1^, µa=0.011mm^-1^] (10). Eq. 14 was used to calculate the expected contrast vectors for these six regions of interest, which is then given in S1 Table.

**Supplemental References**

1. Cox RW. AFNI: software for analysis and visualization of functional magnetic resonance neuroimages. Computers and Biomedical research. 1996;29(3):162-73.

2. Huppert TJ. Commentary on the statistical properties of noise and its implication on general linear models in functional near-infrared spectroscopy. NEUROW. 2016;3(1):010401-. doi: 10.1117/1.NPh.3.1.010401.

3. Barker JW, Aarabi A, Huppert TJ. Autoregressive model based algorithm for correcting motion and serially correlated errors in fNIRS. Biomed Opt Express. 2013;4(8):1366-79. doi: 10.1364/BOE.4.001366. PubMed PMID: 24009999; PMCID: PMC3756568.

4. Beaton AE, Tukey JW. The fitting of power series, meaning polynomials, illustrated

on band-spectroscopic data,. Technometrics. 1974;16(2):147-85.

5. Arridge SR. Optical tomography in medical imaging. Inverse problems. 1999;15(2):R41.

6. Dehghani H, Eames ME, Yalavarthy PK, Davis SC, Srinivasan S, Carpenter CM, Pogue BW, Paulsen KD. Near infrared optical tomography using NIRFAST: Algorithm for numerical model and image reconstruction. Communications in numerical methods in engineering. 2009;25(6):711-32.

7. Fang Q. Mesh-based Monte Carlo method using fast ray-tracing in Plücker coordinates. Biomedical optics express. 2010;1(1):165-75.

8. Holmes CJ, Hoge R, Collins L, Woods R, Toga AW, Evans AC. Enhancement of MR images using registration for signal averaging. J Comput Assist Tomogr. 1998;22(2):324-33. PubMed PMID: 9530404.

9. Tzourio-Mazoyer N, Landeau B, Papathanassiou D, Crivello F, Etard O, Delcroix N, Mazoyer B, Joliot M. Automated anatomical labeling of activations in SPM using a macroscopic anatomical parcellation of the MNI MRI single-subject brain. Neuroimage. 2002;15(1):273-89.

10. Jacques SL. Optical properties of biological tissues: a review. Physics in medicine and biology. 2013;58(11):R37.
